# Mesenchymal Stem Cells Applications in Alzheimer's Disease

**DOI:** 10.1055/s-0043-1777087

**Published:** 2023-12-11

**Authors:** Oluwatosin Debola Oyebode, Pınar Tulay

**Affiliations:** 1 Department of Medical Genetics, Faculty of Medicine, Near East University, Nicosia, Cyprus; 2 DESAM Research Institute, Near East University, Nicosia, Cyprus; 3 Center of Excellence, Genetics and Cancer Diagnosis-Research Center, Near East University, Nicosia, Cyprus

**Keywords:** Alzheimer's disease (AD), mesenchymal stem cells, Aβ plaques, microglia, neuroinflammation

## Abstract

Alzheimer's disease (AD) is a neurodegenerative disorder that advances gradually and primarily impacts the hippocampus region of the brain. It is defined by a deterioration in cognitive function as well as an observable loss of memory retention. One of the major characteristics of AD is the impairment of neural generation, resulting in the depletion of neurons and synaptic connections within the nervous system. It is unfortunate to say that, at present, no definitive cure is available for AD, and no medication is effective in halting the progression of neurodegeneration associated with it. Nevertheless, it is crucial to highlight that progress has been achieved in addressing the troubling symptoms of AD. The Food and Drug Administration has granted approval for two categories of medications designed to alleviate these symptoms. The scientific community has been inspired by these advancements to investigate alternative therapeutic options, with an emphasis on stem cell therapy in particular. The main focus of this review will be on the potential for the use of a variety of mesenchymal stem cells as a treatment for AD.

## Introduction

Alzheimer's disease (AD) stands as one of the prevalent types of dementia, with an estimated 50 million individuals affected globally. Taking into account that age plays a pivotal role in its occurrence and considering the accelerated aging of the population, it is projected that approximately 152 million individuals will be afflicted by AD by the year 2050. 1 Memory loss, disorientation, cognitive decline, inability to carry out daily tasks, and behavioral disturbance are some of the characteristics of dementia. Significantly, AD is one of our era's most significant economic, social, and medical challenges. 2

Sporadic AD arises from a complex interplay of factors, encompassing both genetic and environmental elements. 3 Most cases of AD result from delayed onset and sporadic, and other confirmed risk factors apart from age are cardiovascular disease, depression, limited education, and the ApoE4 (apolipoprotein-E4) gene. 2 PSEN1, PSEN2, and sometimes amyloid precursor protein (APP) gene autosomal mutations account for around 5% of familial AD cases. 2 3

The causes and effects of AD vary but can be characterized by four pathological findings. The first is the hyperphosphorylation of a protein called tau, associated with intracellular microtubules within the neurons essential for supporting structurally and axonal transport. The hyperphosphorylation leads to the collapse of the microtubule and abnormal accumulation of the tau protein within neurons. 3 4 The second one is when amyloid β (Aβ) plaques formed as a result of β- and γ-secretase enzymatic cleavage of the APP, which is a protein found in the neuron and is transmembranal accumulates in the brain. 2 4 Decreasing production by inhibition of secretase or by vaccination has been used as a pharmacological approach to clear the amyloid accumulation. 2 The third hallmark of AD is the activation of microglia, which are specialized immune cells located within the central nervous system (CNS). They can be seen at the beginning of the disease, but their proportion in the brain decreases as AD progresses. Tumor necrosis factor (TNF)-α, interleukin (IL)-1β, and nitric oxide are some of the cytokines produced by the activated microglia, and they cause neuroinflammation. 3 4 Widespread neuronal and synaptic impairment is the fourth pathological characterization of AD. Multiple neurotransmitters are involved in the emergence of AD. In AD, the cholinergic system, which is crucial for cognition, is disrupted, which causes cholinergic neurons to degenerate and the development of amyloid plaque and neurofibrillary tangles. Other neurotransmitter systems, particularly some noradrenergic, serotonergic, and glutamatergic systems, are also involved in the pathophysiology of AD. GABAergic neurons losing inhibitory control can lead to synaptic damage in AD patients. Generally speaking, the interaction of numerous neurotransmitters is crucial for preserving cognitive function, and AD may worsen if any of these systems are out of balance. 4

## Current Therapies in Treating AD and Challenges

Despite the seriousness of AD, the Food and Drug Administration has only licensed two categories of medications for its treatment: cholinesterase inhibitors and *N* -methyl D-aspartate antagonists. 5

Acetylcholinesterase (AChE) inhibitors are categorized into reversible, irreversible, and pseudo-reversible types. They function by blocking the activity of cholinesterase enzymes like AChE and butyrylcholinesterase, which are responsible for breaking down acetylcholine (ACh). As a consequence, these inhibitors elevate the concentration of ACh in the synaptic cleft. This heightened ACh level has a positive impact on cognitive and neural function, especially during the initial phases of AD. 5 6 Examples of these cholinesterase inhibitors are donepezil (it increases the level of AChE by binding to AChE reversibly, thereby inhibiting the hydrolysis of acetylcholine), rivastigmine (pseudo-irreversible inhibitor that inhibits AChE and butyrylcholinesterase which breaks down acetylcholine in the brain), and galantamine (a dual-action tertiary isoquinoline alkaloid, it acts as a competitive inhibitor of AChE by binding allosterically to nicotinic acetylcholine receptors and activating them). 5 7

The pathophysiology of AD is greatly affected by the *N* -methyl D-aspartate receptor (NMDAR) as the stimulation of NMDAR causes Ca ^2+^ influx which triggers transduction of signal, and results in gene transcription necessary for long-term potential formation, which is vital for memory formation, synaptic neurotransmission, and plasticity. Excessive activation of NMDAR leads to an overstimulation of glutamate, which is the primary excitatory neurotransmitter in the CNS. This overstimulation, in turn, causes excitotoxicity, disrupts synaptic function, triggers neuronal cell death, and ultimately results in a decline in cognitive function. 5 8 Memantine, a low-affinity noncompetitive NMDAR antagonist, is currently the only licensed medicine for treating moderate to severe AD in this category. 7 9

The setback with these drugs is that they focus mostly on managing the symptoms but not curing the disease. Also, some of the drugs have adverse side effects, for example, when NMDAR activation becomes excessive it causes abnormal calcium signaling and overstimulation of the neurotransmitter glutamate, which is essential for brain function. This excessive activity leads to excitotoxicity, disrupted synaptic function, death of nerve cells, and decline in cognitive abilities. 5 7

The main challenge lies in developing effective disease-modifying treatments that can slow or stop the progression of Alzheimer's. Researchers are actively working on various approaches to address this challenge and provide better options for patients in the future, among this is the treatment with mesenchymal stem cells (MSCs).

## Mesenchymal Stem Cells Applications in AD

MSCs are spindle-shaped, adhere to plastic, migrate to the injury site, and are multipotent. They can be obtained from sources but are not limited to the bone marrow, adipose tissue, and Wharton's jelly. In recent years, their multipotency ability, immune system modulator property, and neurotrophic functions have drawn much attention to using them as therapeutic tools. 10

When treating AD with MSCs, MSCs proliferate astrocytes, metabolize glutamate and gamma-aminobutyrate, inhibit neural cell necrosis, and unleash growth factors (like brain-derived neurotrophic factor [BDNF]) to enhance neurogenesis by stimulating neural progenitor cells because of their antioxidant properties and antiapoptotic effects. 11 12 To stop additional tissue damage brought on by chronic neuroinflammation, MSCs also have modulatory effects on the immune system by bypassing or suppressing proinflammatory microglia (M1) activation and encouraging the activation of anti-inflammatory microglia (M2). Research has proven that MSCs can boost autophagy activation, which is likely to be the reason for the lysosomal clearance of Aβ plaques. MSCs also speed up the buildup of microglia near Aβ deposits to facilitate Aβ. 12

## Autophagy and Apoptosis in AD: Bone Marrow-Derived Mesenchymal Stem Cell Role in Transplantation

### Autophagy and Bone Marrow-Derived Mesenchymal Stem Cells Effect

Aβ peptide removal and tau protein assembling in cerebral tissue is a function of autophagy. The cytoplasm components are sequestrated into autophagosomes for subsequent degradation and recycling by autophagy. Given that accumulation of abnormal Aβ peptide is a hallmark of AD, when autophagy is deregulated, AD progresses because functioning autophagy reduces neuropathology as seen by molecular markers like Beclin-1, atg7, Lamp-1, Lamp-2, and mammalian target of rapamycin (mTOR) being expressed. 13 14 15 mTOR complex major is correlated with removing Aβ proteins via regulating the primary signaling pathway PI3k/AKt, GSk-3, AMPk, and IGf-1. 15 16

Aberrant mitophagy and the ensuing dysfunctional Aβ and tau pathology show how autophagy malfunctions in AD development. In animal models and people with sporadic late-onset AD, reduced mitophagy is associated with synapse deterioration and cognitive impairments. 15 17

Behavioral and cognitive improvements are associated with enhancing autophagy. In AD-like models, after the transplant of bone marrow-derived MSCs (BMMSCs), reduced amounts of abnormal Aβ and hyperphosphorylated tau proteins lower the death of the neuron. In the hippocampus, LC3-II-positive autophagosomes are upregulated as well as the BECN1/Beclin 1 secretion which activates the Aβ peptides clearing in AD-like models. 15 Furthermore, after the transplant of BMMSCs, a variety of cytokines are released within the local microenvironment through both autocrine and paracrine signaling mechanisms. 18

## Apoptosis and Bone Marrow-Derived Mesenchymal Stem Cells

BMMSCs transplantation can restore the damage caused by the mechanism of apoptosis (programmed cell death), which results in the death of neurons and loss of memory in AD animal models. Activating nuclear factors such as p53, Foxa2, and C/EBP, increasing antiapoptotic proteins like B cell lymphoma-2 and survivin, and indirectly controlling signal molecules like stromal cell-derived factor-1 and neurotrophic growth factor are some of the levels at which the apoptosis signaling cascade can be modulated. 15

The effects of BMMSCs transplantation on apoptosis can be direct or indirect. Direct effects include the inhibition of caspases, a family of proteins involved in the programmed cell death pathway, through antiapoptotic Bcl-2 and increased expression of survivin and seladin-1. 15 18 19 BMMSCs can additionally upregulate the production of antiapoptotic proteins belonging to the inhibitor of apoptosis proteins (IAPs) family, such as XIAP. This augmentation effectively inhibits neuronal apoptosis, providing neuroprotective effects. 15 20

Indirect effects of BMMSCs transplantation include the elimination of Aβ peptides, which accumulate in AD and trigger apoptosis via regulators such as stress-activated protein kinases and p53 expression. 15 18 BMMSCs can also reduce apoptosis by induction of mitophagy, which eliminates oxidized materials and abnormal proteins, and by stimulating the endogenous antioxidant system. The other effects of BMMSCs transplantation include the production of cytokines and neurotrophic factors that promote angiogenesis and neurogenesis. 15

## Adipose Tissue-Derived MSCs Beneficial Effects on Microglia for AD Treatment

Adipose tissue-derived MSCs (AD-MSCs) are gotten from adipose tissue (belly fat, buccal fat pad, infrapatellar fat pad), stick to plastic, exhibit surface markers (like CD29, CD44, CD105), and also lack some surface markers (like CD14, CD19, CD45). 21

According to studies, AD-MSCs can promote endogenous neuron growth in the subgranular and subventricular zones, decrease oxidative stress, and alleviate mental impairment in APP/PS1 mice. In animal models, AD-MSCs also improve AD symptoms by regulating inflammatory mediators, microglia proliferation, polarization, and phagocytic activity. 22

In a brain in good condition, microglia maintain the homeostasis of the CNS. Microglia support the CNS by secreting neurotrophic factors and responding to alterations in the brain by clearing up cellular debris. When there is an injury in the brain or persistent triggers, microglia can produce excessive inflammation that can harm the brain. 21

The ability of the microglia to switch from proinflammatory (with a round morphology in vitro) to neuroprotectiveness (elongated shape) makes it an excellent therapeutic focus in neuroinflammatory diseases because it aims to maintain microglia in a neuroprotective state that supports the CNS by influencing their inflammatory condition. Aβ accumulation (a pathological hallmark of AD) can trigger the proinflammatory state. Reactive oxygen species, nitric oxide, and some proinflammatory cytokines like TNF-α, IL-1β, and IL-6 secreted are what start the proinflammatory phenotype. 21 23 Studies show MSCs and IL-4 can produce the neuroprotective phenotype. However, this phenotypic induction has not received as much attention as its proinflammatory counterpart. BDNF, activity-dependent neurotrophic protein (ADNP), and the fractalkine receptor CX3CR1 are neuroprotective substances upregulated in this phenotype. 21

In vitro, under inflammation, primary microglia will have a round shape similar to in vivo; when CNS microglia undergo pathological events, the microglia will have an amoeboid morphology. When AD-MSCs were incubated with the microglia from a primary mouse (living mouse without prior passaging or manipulation in a laboratory setting) in vitro, the microglia changed their morphology drastically to an elongated cell shape. This incorporation prevents direct cell-to-cell interaction; therefore, the shape change must result from soluble factors. The conditioned medium (CM) from AD-MSC also causes this change in morphology. The change in morphology shows that when AD-MSC or AD-MSC-CM are incorporated in vitro, the inflammatory phenotype is reversed into a neuroprotective one. 21 This switch causes a reduction in the proinflammatory cytokines released and increases neuroprotective factors like BDNF, ADNP, fibroblast-growth factor-2, and arginase-1, a marker that activates macrophages and phagocytosis. This increase is significant because the mouse model for AD shows that phagocytosis reduces as Aβ increases. 21 22

## Targeting Microglial Signaling Pathways for Anti-Inflammatory Therapy

Specific signaling pathways in microglia can influence whether they display a proinflammatory or anti-inflammatory neuroprotective phenotype. The activation or inhibition of particular proteins within these pathways could be a potential target for drug development to shift microglia toward an anti-inflammatory state. The presence of cerebrospinal fluid-1 (CSF-1) has been identified in conditioned media from AD-MSC-CM. When mice lose their CSF-1 receptor (CSF-1R), they experience a significant reduction in microglia, indicating the crucial role of CSF-1 in microglial survival pathways. In vitro experiments using anti-CSF-1 antibodies have shown that CSF-1R signaling may play a part in the process by which AD-MSC-CM influences microglia branching. Additionally, downstream targets of CSF-1R in macrophages, such as PI3K and PKB/Akt, are likely involved in the microglia branching induced by AD-MSC-CM. Once activated, PI3K generates PIP3, which activates RhoGTPases, including RhoA, Rac1, and Cdc42, the primary controllers of the actin cytoskeleton responsible for morphological changes in cells. In microglia, Rac1 activation induces lamellipodia formation, which is detectable by microscopy and associated with microglial ramification. 21 24

## Exposure to AD Environment Enhances Therapeutic Effects of WJ-MSCs (Wharton's Jelly- MSCs) Against AD Traits In Vitro and In Vivo

Under serum starvation, primed MSC had an antiapoptotic impact on the H4 Swedish cell line. WJ-MSC was cocultured with an AD cell line to obtain a primed MSC. H4SW cells and the primed MSCs were grown together for 24 hours. The results revealed that after being serum-starved for 24 hours, H4SW cells underwent apoptosis. In contrast, apoptosis was stopped when naive MSCs and primed MSCs were cultured with the H4SW cells, although the primed MSCs group showed the highest antiapoptotic effects. Also, cleaved poly(ADP-ribose) polymerase and caspase-3 cell death markers were seen to be reduced in the cocultured cells. 25

Accumulated Aβ and ubiquitin conjugates are the prominent pathological findings of AD. 2 25 To test the therapeutic impact against AD characteristics, primed MSCs were cultured with H4SW in vitro for 24 hours. After the culturing, enzyme-linked immunosorbent assay was used to check the Aβ level, and it was found that the Aβ secreted reduced drastically compared with the H4SW cell control. Also, in the cytosol, more ubiquitin conjugates were accumulated in the AD in vitro model (H4SW), but in the primed and naive MSCs, ubiquitin conjugates were reduced significantly. 25

An in vivo study looked at the efficacy of primed MSCs in treating AD in a transgenic mice model termed 5xFAD. The experimental mice, separated into four groups at 12 months old including wild-type control (WT), transgenic control, naive-MSC, and primed MSC, underwent injection of 1 × 105 WJ-MSCs into the right lateral ventricle. The mice were killed after a week, and the brain tissue was harvested. By using cleaved caspase-3 Western blot analysis, the antiapoptotic effect of primed MSCs was evaluated. In the brains of the 5xFAD animals, cleaved caspase-3 levels were greater than those of WT mice, indicating neuronal death. However, the cleaved caspase-3 in the brain was dramatically decreased by the naive and primed MSCs. 25

## Therapeutic Effect of Exosomes-Derived from MSCs

Exosomes, also known as small extracellular vesicles (EVs), are released by several cell types. They vary in size from 30 to 150 nm and might reflect the condition of the parent cell. 26 27 EVs can traverse the blood-brain barrier and facilitate the transfer of proteins, lipids, and nucleic acids from one cell to another in response to specific physiological cues to which these cells are exposed. 27 28

Aβ plaques are formed as a result of β- and γ-secretase enzymatic cleavage of the APP. 2 4 In contrast to oligomers, Aβ monomers are harmless. The accumulation of Aβ is imbalanced in AD. Pathogenic proteins spread through the exosome pathway once lysosomes or glial cells are overloaded. Treatment of AD involves clearing pathogenic proteins through NEP (neprilysin) and IDE (insulin-degrading enzyme) activity. Exosomes derived from MSC **s** with NEP and IDE activity reduce Aβ plaques in transgenic mice, indicating the potential for treating AD. 27 29

Substantial evidence supports the idea that the immune system plays a pivotal role in the development of AD. To our understanding, membrane interactions affect traditional neuroimmune cell-to-cell communication. Due to low concentrations of costimulatory molecules and class II major histocompatibility complex on the cell surface, MSCs have an immunomodulatory function. It is important to highlight that MSC-exos can control immune cells since they include immunologically active chemicals. For instance, MSC-exos help prevent lymphocyte proliferation and differentiation. 27

In addition, exosomes that are derived from MSC **s** encourage the development of lymphocyte subtypes that are less likely to cause inflammation. According to what we know, MSC-exos can enhance regulatory cell expression, reduce the ability of T cells to differentiate into IL-17-producing effector T cells, and promote the differentiation of Th 1 cells into Th 2 cells. 30 Numerous findings have revealed that the inflammatory cytokines and proteins included in MSC-exos have immunomodulatory capabilities. 31

A crucial pathogenic mechanism in AD is neuroinflammation. 3 Neuroinflammation was sparked by an excessive buildup of Aβ in the brain. MSC-exos dramatically enhances spatial learning ability and memory impairment in AD transgenic mice by regulating the immune system and reducing neuroinflammation in pathological aberrant regions. Additionally, by inhibiting the production of cytokines, reactive astrocytes, and activated microglia, MSC-exos also contribute to creating anti-inflammatory effects. 27 28

In Alzheimer's, synapse dysfunction is a common early-stage symptom leading to cognitive impairment. 4 According to recent studies, human MSC exosomes (hMSC-EVs) can prevent oxidative stress and Aβ oligomer (AßO)-induced synapse degradation to protect hippocampal neurons. This neuroprotective effect is related to catalase, an active antioxidant enzyme, in the exosomes. Synaptic protein expression, such as Synapsin 1 and PSD95, reflects how synapses function in various aspects. These synaptic proteins are expressed more highly when MSCs are subjected to hypoxia, which enhances synaptic function. Exosomes obtained from astrocytes reduce the amounts of apoptotic proteins and increase neurite growth, suggesting a potential clinical treatment for AD. MSC-exos can also transfer miR-133b into astrocytes and neurons, promoting the restoration of brain function. 27

Exosomes are also being explored as a promising drug delivery vehicle in treating AD. Exosomes also can move active molecules between cells, are biocompatible, and have a blood-brain barrier crossing capacity. They have low immunogenicity and a lengthy half-life in circulation, which helps delay the quick deterioration of “therapeutic cargo.” The spleen and liver appear to be the primary locations of intravenously delivered exosomes, with the brain showing weaker signals. By connecting peptides, the surface of the exosome can be modified to address this issue, thereby increasing their concentration in the brain. Exosomes derived from dendritic cells that have been engineered to express the membrane protein Lamp2b has shown the ability to bind to the rabies virus glycoprotein peptide, a feature specific to neurons. When these engineered exosomes, known as MSC-exos, were administered to transgenic mice with AD, they were observed to have a substantial positive impact on cognitive function, significantly improving it. 25 27 32

## Challenges and Perspective

Although the safety of MSCs has been established through clinical trials, their efficacy is yet to be determined. The fact that AD has already caused neuronal death and aberrant proteins to collect in many different brain locations by the time it is identified presents one of the treatment hurdles. Additionally, most clinical trial protocols involve patients receiving only a few stem cell infusions despite needing several over an extended period.

Intravenous infusion of MSCs has been ineffective, as only a small percentage can enter the brain while most are retained in other organs. Autologous MSCs may experience senescence due to elderly age, hampering their regeneration capacity. 1

There is a link between autophagy activation and neuronal survival but inconsistent outcomes. Using autophagy-related medications alone may not be sufficient to stop advanced AD from progressing. 15

BMMSC transplantation could develop into neurons important for synaptogenesis and enhancing cognitive function; however, there is uncertainty about how long this balance will remain effective. 18 Integrating various mechanisms, including immune system regulation, prevention of cell death, stimulation of nerve cell growth, enhancement of cellular self-cleansing processes, and promotion of blood vessel formation, may offer therapeutic advantages for individuals with AD. 15 18

In addition, immunomodulation and neuroprotection mediated by exosomes are comparable to those mediated by transplanted stem cells, but additional research is necessary. 18

## Conclusion

Finally, MSCs and their exosomes have shown tremendous therapeutic promise for AD through various activities, including immunoregulation, antiapoptosis, neurogenesis, autophagy activation, and angiogenesis. The protection of hippocampal neurons, enhancement of synaptic function, and transfer of miR-133b into astrocytes and neurons by exosomes produced from MSCs have all been linked to improvements in cognitive performance. Determining the effectiveness of MSCs and solving problems like the restricted brain penetration of exosomes given intravenously remain challenging tasks. A more comprehensive investigation is required to fully grasp the potential of MSCs and their exosomes in the treatment of AD.
